# Epidemiological distribution of MRI‐based presumptive imaging diagnoses in a large general population of cats with suspected epileptic seizures

**DOI:** 10.1002/vetr.70737

**Published:** 2026-05-09

**Authors:** Anna Knebel, Sebastian Meller, Julien Labruyère, Holger A. Volk

**Affiliations:** ^1^ Department of Small Animal Medicine & Surgery University of Veterinary Medicine Hannover Hannover Germany; ^2^ Center for Systems Neuroscience Hannover (ZSN) Hannover Germany; ^3^ VetCT Cambridge UK

## Abstract

**Background:**

The reported prevalence of feline idiopathic epilepsy (IE) and presumptive imaging diagnoses based on magnetic resonance imaging (MRI) varies depending on factors such as differences between primary care versus referral study populations.

**Methods:**

This retrospective, imaging‐based study reviewed MRI data from cats with suspected epileptic seizures. Cases were categorised as follows: no lesions detected (IE, referring to cats aged 6 months‒7 years with recurrent epileptic seizures, and epilepsy or epileptic seizure of unknown aetiology [EuA] [i.e., cats not fulfilling IE criteria]) and structural lesion(s) detected (SL). Presumptive imaging diagnoses were classified according to the following aetiologies: presumptive intra‐axial/extra‐axial neoplasia, inflammatory, vascular, congenital, traumatic, metabolic/toxic and degenerative.

**Results:**

Of 432 MRIs, 39% revealed SL, whereas 61% showed no structural lesions (of which 47% of cases were classified as IE and 53% were classified as EuA). Among cats with SL, presumptive imaging‐based diagnoses included inflammatory diseases (25%), extra‐axial neoplasia (19%), metabolic/toxic aetiologies (12%), congenital disorders (10%), lesions of unknown relevance (9%), intra‐axial neoplasia (7%), trauma (5%), suspected hippocampal necrosis (5%), vascular events (4%), thiamine deficiency (2%) and degenerative diseases (2%).

**Limitations:**

This imaging‐based study lacked histopathological confirmation and comprehensive clinical information.

**Conclusion:**

Nearly two‐thirds of MRIs involving cats with suspected epileptic seizures showed no structural brain abnormalities.

## INTRODUCTION

Approximately 2%‒3.5% of the feline population experiences recurrent epileptic seizures,^1^, ^2^, ^3^, ^4^ which results from an imbalance between inhibitory and excitatory neurotransmitters, leading to paroxysmal, hypersynchronous and generally self‐limiting neuronal activity.^2^, ^4^, ^5^, ^6^, ^7^ Emergency situations such as status epilepticus (epileptic seizure lasting >5 minutes or multiple discrete epileptic seizures without recovery of consciousness) and cluster seizures (≥2 epileptic seizures within 24 hours) can occur when seizure activity persists unabated.^5^

Various causes can lead to reactive seizures, including intoxications, metabolic disturbances and underlying medical conditions.^2^, ^5^, ^8^ Epilepsy is characterised by at least two unprovoked epileptic seizures more than 24 hours apart and is broadly classified aetiologically as idiopathic epilepsy (IE) or structural epilepsy (SE).^3^, ^5^ SE results from detectable brain abnormalities, such as ischaemic or haemorrhagic infarcts, inflammatory lesions (infectious or immune mediated), neoplasia, traumatic injuries, congenital malformations or degenerative changes.^2^, ^9^ IE remains a diagnosis of exclusion, defined by a normal clinical and neurological examination during the interictal period and diagnostics including blood analysis (complete blood count and biochemistry profile), urinalysis, liver function tests (pre‐ and post‐prandial serum bile acids or ammonia tolerance test), T4, infectious disease screening (e.g., *Toxoplasma gondii*), magnetic resonance imaging (MRI) and cerebrospinal fluid (CSF) analysis being within normal limits.^1^, ^2^, ^3^, ^5^, ^8^, ^10^, ^11^ Approximately 25%‒54% of cats with epilepsy are reported to have IE.^1^, ^2^, ^3^, ^4^, ^8^, ^9^, ^10^, ^11^, ^12^, ^13^ A greater suspicion of SE is associated with epileptic seizures developing in cats over 7 years of age, especially where the neurological deficits are asymmetrical.^3^, ^5^, ^11^ In cases of epilepsy of unknown aetiology (EuA), the underlying cause remains undetermined. However, considering factors such as advanced age, IE is deemed less likely.^1^, ^9^, ^14^

Unfortunately, epileptic seizure recognition in cats can be challenging due to subtle and variable manifestations, such as orofacial twitching, excessive salivation and psychomotor activity (hyperactivity, mydriasis and aggression).^8^, ^15^ Distinguishing between IE and SE often requires advanced diagnostic modalities, such as MRI of the brain and comprehensive CSF analysis.^10^ However, there are circumstances where such advanced diagnostic tests cannot be performed due to logistical, financial or technical constraints. Consequently, the reported prevalence of suspected IE and SE in the veterinary literature demonstrates considerable variability, reflecting differences in study design, sample size and the characteristics of study population sourced from primary care versus referral centres.^1^, ^2^, ^3^, ^16^, ^17^

Similar to a prior MRI‐based retrospective study involving dogs with epileptic seizures,^18^ this retrospective imaging‐based study aimed to analyse the epidemiological distribution of MRI‐based presumptive imaging diagnoses in cats with structural brain lesions and to provide prevalence estimates of IE in a large referral cohort of cats undergoing MRI for suspected epileptic seizures.

## MATERIALS AND METHODS

### Case identification

A large database of feline brain MRI images and corresponding reports written by board certified veterinary radiologists, provided by the teleradiology company VetCT, was reviewed. This approach was chosen to increase case numbers and ensure inclusion of non‐referral cases, thereby reflecting the typical clinical caseload encountered in daily veterinary practice. This database comprised feline brain MRI studies performed between 1 January 2010 and 27 December 2019. Cats were selected according to the following search terms within the case history: ‘cat’, ‘seizure(s)’, ‘convulsion(s)’, ‘fit(s)’, ‘fitting’, ‘fitted’, ‘epilepsy’, ‘focal epilepsy’, ‘(facial) twitching’, ‘episode(s)’, ‘status’, ‘cluster’, ‘drooling’, ‘mydriasis’, ‘licking fit(s)’, ‘running (fits)’, ‘fly‐biting’, ‘tick(s)’, ‘shaking’, ‘absence’ and ‘seizuring’. Cases in which terms such as ‘convulsion’, ‘seizuring’ or ‘fit’ were mentioned without further clarification regarding epileptic seizure recurrence (potentially representing a single witnessed epileptic event) were also included in this study.

For inclusion in the study, MRI protocols of the brain required a minimum of T1‐weighted (T1w) and T2‐weighted (T2w) sagittal and transverse sequences, with additional sequences (e.g., T1w pre‐ and postcontrast dorsal, sagittal and transverse sequences, transverse and/or dorsal or sagittal fluid‐attenuated inversion recovery [FLAIR] sequence, gradient and spin echo sequence, and susceptibility‐weighted image and diffusion‐weighted image sequence) included when available.

MRI studies and the corresponding reports from VetCT's database were reviewed in addition to the following information, if available: age, breed, sex, neuter status, clinical history, general and neurological examination findings, blood analysis (complete blood cell count), biochemical profile (liver biochemistry profile ± pre‐ and post‐prandial serum bile acids or ammonia, kidney biochemistry profile and electrolytes), urinalysis, infectious disease testing (feline leukaemia virus [FeLV], feline immunodeficiency virus [FIV] and *T. gondii*) and CSF analysis.

Using the information available from this database, including imaging studies, descriptions and diagnostic results, cats were further categorised according to the criteria outlined in Table 1 (presumptive imaging diagnosis based on imaging characteristics). Cases were excluded if further details suggested another type of episode such as vestibular disease.

**TABLE 1:** Definitions of the groups cases were categorised into.

Main group	Definition of main group	Subgroup	Definition of subgroup
No structural lesion(s)	No significant structural brain lesion(s) detected on MRI or a structural brain lesion(s) detected but determined by radiologists to be highly unlikely to cause epileptic seizures	IE—idiopathic epilepsy	No relevant lesion(s) on MRI detected as well as at least two epileptic seizures at least 24 hours apart. Cats 6 months‒7 years of age.^3^, ^10^, ^11^
		EuA—epilepsy or epileptic seizure of unknown aetiology	No relevant lesion(s) on MRI detected but IE is less likely due to recurrent epileptic seizures occurring outside the defined age range for IE, or cases with unconfirmed seizure recurrence.
Structural lesion(s)	Structural brain lesion(s) detected on MRI, which are determined to be the likely cause of epileptic seizures and categorised according to the top differential diagnosis by the reporting radiologist (based on MRI findings and characteristics)	V—vascular disease	Presumptive imaging diagnosis of vascular accident considered most likely by reporting radiologist.^19^, ^20^, ^21^, ^22^, ^23^
		IA—intra‐axial neoplasia (intra‐axial lesion with characteristics most consistent with IA)	Presumptive intra‐axial mass lesions compatible with an imaging diagnosis of IA considered most likely by reporting radiologist (inflammatory lesions cannot be completely excluded due to lack of histopathology).^19^, ^24^, ^25^, ^26^
		EA—extra‐axial neoplasia (extra‐axial lesion with characteristics most consistent with EA)	Presumptive extra‐axial mass lesions compatible with an imaging diagnosis of EA considered most likely by reporting radiologist (inflammatory lesions cannot be completely excluded due to lack of histopathology).^19^, ^24^, ^25^, ^27^
		I—inflammatory disease (intra‐ and/or extra‐axial lesions most consistent with inflammatory disease)	Presumptive inflammatory disease considered most likely by reporting radiologist (neoplastic lesions cannot be completely excluded due to lack of histopathology).^19^, ^26^, ^28^, ^29^, ^30^ Suspected HN was evaluated separately on account of specific imaging characteristics. Cats with imaging findings compatible with the presumptive pyogranulomatous form of feline infectious peritonitis were included within this group (based on imaging findings, histopathological confirmation not available).^19^, ^29^
		HN—hippocampal necrosis	Imaging findings compatible with presumptive HN considered most likely by reporting radiologist (based on imaging findings, histopathological confirmation not available).^14^, ^31^, ^32^
		C—congenital malformation	Imaging diagnosis of presumptive C considered most likely by reporting radiologist (histopathological confirmation not available).^19^, ^33^, ^34^
		D—degenerative disease	Imaging diagnosis of a presumptive D is considered most likely by reporting radiologist (histopathological confirmation not available).^19^, ^35^, ^36^, ^37^, ^38^, ^39^, ^40^, ^41^
		MT—metabolic/toxic cause	Imaging findings compatible with suspected toxic or metabolic aetiology (other causes such as D cannot be excluded due to lack of histopathology).^19^, ^42^, ^43^
		TD—thiamine deficiency	Imaging diagnosis of presumptive TD is considered most likely by the reporting radiologist, based on MRI description/findings (histopathology and blood thiamine concentrations not available).^19^, ^44^, ^45^, ^46^
		T—trauma	Evidence of traumatic injury on MRI (no histopathology).^19^, ^47^
		U—unknown relevance	Structural brain lesion(s) detected on MRI but with uncertain clinical significance as a cause/reason for epileptic seizures (e.g., signal changes within the frontal sinus or masses in this region but unclear connection to the brain/suspected intact cribriform plate or mild ventriculomegaly of unknown aetiology).

Abbreviation: MRI, magnetic resonance imaging.

For the purposes of this study, the term ‘structural lesion(s)’ (SL) was used in place of SE to encompass a broader spectrum of cases, including those with presumed metabolic or nutritional (thiamine deficiency) aetiologies.

Age‐based stratification of cases was performed as follows^3^, ^7^—group 1: less than 1 year; group 2: 1‒7 years; group 3: greater than 7 years.

### Data analysis

Microsoft Excel 2022 (Microsoft Corporation) was used for data collection, data analysis (descriptive data) and creation of figures. Prism 10 software (GraphPad) was used for further statistical analysis. A chi‐squared test was used to investigate the association between age (groups 1–3) and MRI changes (unremarkable MRI or MRI with structural abnormalities). A Fisher's exact test and multiple logistic regression analyses were used to investigate associations between neurological examination findings and the likelihood of pathological changes being detected on MRI. The logistic regression model included the MRI studies as binary dependent variables (remarkable vs. unremarkable), neurological examinations as independent binary variables (remarkable vs. unremarkable) and age groups as independent categorical variables (groups 1–3).

## RESULTS

### Study population

A total of 1465 imaging studies of feline brain MRIs (some also including parts of the cervical spinal column) were available in the VetCT database for review. MRIs of cats with presenting complaints involving vestibular/ear disease, ataxia/tetraparesis, cranial nerve deficits, respiratory/upper airway problems, blindness, swellings, collapse and trauma were excluded. Using the aforementioned search terms, 432 cats were included in this study.

The study population consisted of the following breeds: Domestic Shorthair (*n* = 275), Domestic Longhair (*n* = 31), British Shorthair (*n* = 21), Crossbreed (*n* = 16), Maine Coon (*n* = 10), Siamese (*n* = 9), Birman (*n* = 8), other (*n* = 8), Norwegian Forest (*n* = 7), Ragdoll (*n* = 6), Persian (*n* = 6), Exotic Shorthair (*n* = 4), Siberian (*n* = 3), Burmese (*n* = 3), Bengal (*n* = 3) and two of each of the following breeds: Egyptian Mau, Chinchilla (British Chinchilla), Russian Blue, Sphynx and Abyssinian. One cat of each of the following breeds was also included in the study population: Scottish Shorthair, Ocicat, Scottish Fold, American Shorthair, LaPerm, Turkish Van, Devon Rex, Toyger, Cornish Rex, Manx, Turkish Angora and Oriental Shorthair. Of all the cats, approximately 40% were male neutered (*n* = 173), 32.6% were female neutered (*n* = 141), 13.9% were female entire (*n* = 60), 11.6% were male entire (*n* = 50) and for eight cats, the sex was not available (1.9%). The mean age of the whole study population was 85 months (1‒222 months), with 46% (*n* = 198) aged 1–7 years and 45% (*n* = 194) aged over 7 years. Only 9% (*n* = 40) were younger than 1 year.

General clinical and neurological examination data were available for 35% and 53% of cases, respectively. Haematology and biochemistry results (liver and kidney biochemistry profile and electrolytes) were available for 40% of cases. Results of urinalysis and infectious disease testing (FIV, FeLV and *Toxoplasmosis*) were available for 4% and 11% of the cats, respectively. Specific details on the types of assays performed for screening for FeLV/FIV and *T. gondii* were not included in the clinical history submitted, with only reporting of results as positive/negative available for review. CSF analysis was reported in 3%, with all results reported as being unremarkable (Supporting Information).

Of the cats that had complete neurological examination findings available for review (*n* = 229), 87 (38%) had an SL visible on the MRI. Reviewing neurological examination findings for these cats with visible SLs on MRI identified 58 of 87 (67%) with neurological deficits. However, 43 of 142 (30%) cats with epileptic seizures demonstrated neurological deficits, despite an unremarkable MRI study. The likelihood of finding structural changes on the feline brain MRIs in this study was 4.5 times higher in cats with epileptic seizures and concurrent neurological deficits, as long as all other factors in the regression model remained constant. A correlation was found between the age groups (groups 1–3) and MRI results (either unremarkable MRI or MRI with structural changes), showing that cats in group 1 (<1 year) had a higher incidence of structural changes visible on MRI (54%) compared to cats in groups 2 (28%) and 3 (46%). When compared with age group 1, the likelihood of a cat having a structural brain lesion decreased by 72% if it was within age group 2 (1‒7 years of age) and by 24% if it was within age group 3 (>7 years of age); as long as all other factors in the regression model remained constant.

### Magnetic resonance imaging

The study population of 432 cats consisted of 265 cats without detectable structural abnormalities on the MRI (no detectable structural lesion [NL], 61.4%) and 167 cats with detectable structural brain lesion(s) as a possible cause for epileptic seizures (SL, 38.6%). The distribution of presumptive diagnoses in the overall study population is summarised in Figure.1

**FIGURE 1:**
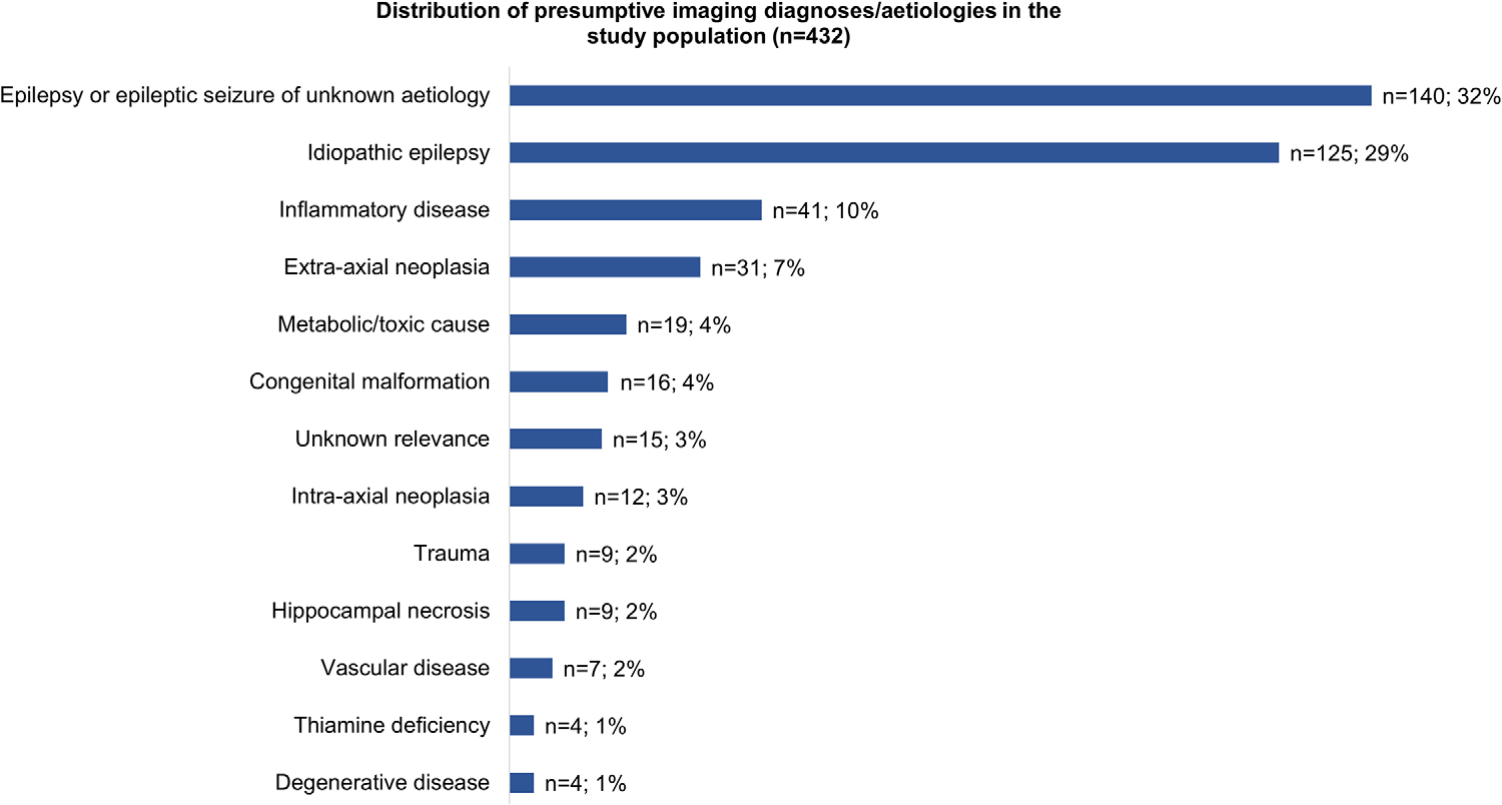
Distribution of presumptive imaging diagnoses/aetiologies for epileptic seizures within the study population (*n* = 432).

IE was suspected in 125 of 265 (47%) cats with an unremarkable MRI scan, and 140 of 265 (53%) cats with unremarkable MRI scans were classified as having epilepsy or epileptic seizure of unknown aetiology (EuA). This latter group included cats with recurrent epileptic seizures, which did not meet the age criteria for IE, in addition to cats within the IE age range but without clearly stated epileptic seizure recurrence.

Focusing on cats with SLs on MRI, presumptive inflammatory lesions and presumptive extra‐axial neoplasia comprised the two largest groups (41/167, 25% and 31/167, 19%, respectively), followed by presumptive metabolic/toxic aetiologies (19/167, 12%) and presumptive congenital aetiologies (16/167, 10%). The remaining cases were characterised as presumptive intra‐axial neoplasia (12/167, 7%), trauma‐related lesions (9/167, 5%), cerebrovascular accidents (7/167, 4%) and neurodegenerative disease (4/167, 2%). In 5% of the cases (9/167), the MRI changes were highly suspicious for feline hippocampal necrosis (HN), and in four cats, the MRI changes were compatible with presumptive thiamine deficiency (4/167, 2%).

In seven of 167 (4%) cats with SL, the MRI findings were consistent with the pyogranulomatous form of feline infectious peritonitis (FIP).^19^, ^29^ Among cats presenting with MRI abnormalities suggestive of extra‐axial mass lesions/presumptive extra‐axial neoplasia, 58% (18/31) exhibited imaging features most consistent with a presumptive diagnosis of meningioma.^19^, ^24^, ^27^, ^48^ In 15 of 167 cats (9%), the clinical relevance of the detected MRI changes as a cause of epileptic seizures was unclear.

## DISCUSSION

This retrospective imaging‐based study analysed MRI findings in a large population of cats with suspected epileptic seizures, revealing that nearly two‐thirds of cats had NLs. IE was suspected in approximately 29% of the overall population of cats, which is consistent with the previously estimated prevalence of IE of 25%‒54%.^1^, ^2^, ^3^, ^4^, ^8^, ^9^, ^10^, ^11^, ^12^, ^13^ Cats in age group 2 (1‒7 years) demonstrated the lowest prevalence of MRI abnormalities, with approximately 28% of cats in this category exhibiting structural brain changes. In contrast, MRI abnormalities were observed in 54% of cats in age group 1 (<1 year) and 46% of those in age group 3 (>7 years).

The results of this study generally align closely with those of Riegler et al. and Marsh et al., who reported that approximately 50% and 75% of epileptic feline patients exhibited unremarkable MRI findings, respectively.^49^, ^50^ Riegler et al. described structural (extrahippocampal) changes, such as neoplasia, in 18% of cats, but found more cats with hippocampal alterations (33%).^49^ The predominant aetiologies within the SE cohort in the Marsh et al. study included traumatic causes (6.5%), degenerative disease (5%), vascular accidents (5%), infectious/inflammatory conditions (3.9%), congenital malformations (2.6%) and neoplastic lesions (1.3%).^50^ Marsh et al.’s study involved a population of cats originating from two referral centres, where comprehensive patient examination and diagnostics could be performed, and therefore data regarding CSF analysis were available in 83% of the cases, FIV/FeLV testing in 66%, *T. gondii* serology in 45% and *T. gondii* CSF PCR in 46%.^50^ However, histopathological confirmation was not available in this study either. By contrast, the current study identified a lower proportion of presumptive degenerative diseases (2%) and a higher prevalence of presumptive inflammatory (25%), congenital (10%) and neoplastic (26%) abnormalities. Traumatic and vascular aetiologies were comparably represented, accounting for 5% and 4% of cases, respectively.

Raimondi et al. reported an even lower prevalence of SE, at approximately 12%, with the authors of this study specifically including cats with epileptic seizures that exhibited no interictal neurological deficits; thereby potentially excluding cases that could have SE.^7^ Recently, a retrospective observational study was published that focused on seizure type and demographic factors. In this study, MRI was not performed in 25.4% of cases, but among cats that did undergo MRI, relatively few animals were found to have structural brain lesions (SE, 15.7%).^51^ Neoplastic disease (not differentiated between presumptive intra‐ and extra‐axial lesions) and inflammatory aetiologies were more frequently suspected.^51^ No vascular, traumatic or degenerative causes were reported,^51^ which aligns with the low numbers in the present study. The generally higher percentages in the current study are likely attributable to the substantially larger sample size. In contrast, one study published in 2010, involving 125 cats with recurrent epileptic seizures, reported that 38% were diagnosed with primary epilepsy (comparable to IE), while the remaining 62% were classified as having secondary epilepsy (comparable to SE).^4^

Focusing on inflammatory disease, a previous study investigating inflammatory aetiologies in feline SE cases identified inflammatory disease in 51.43% of cats (36/70).^52^ FIP was suspected in 5.71% (4/70) of cases, and feline limbic encephalitis was suspected in 12.86% (9/70).^52^ In contrast, the present study found a lower prevalence of presumptive FIP and HN based on imaging findings (1.6% [7/432] and 2% [9/432], respectively). As the presumptive diagnosis relied solely on imaging findings, this prevalence may be underestimated, which could explain the difference in reported numbers.

The prevalence of imaging‐based presumptive HN was also reported to be higher in other studies (6% in Stançiu et al.^3^ and 11% in Pakozdy et al.^4^). Presumptive HN is typically diagnosed based on bilateral symmetrical T2w and FLAIR hyperintensity, T1 hypointensity and moderate to marked contrast enhancement of the piriform lobe and hippocampus (without mass effect), although definitive diagnosis requires histopathological confirmation.^14^, ^31^, ^32^ Moreover, difficulties in interpretation can arise. For example, the piriform lobe may show T2w and FLAIR hyperintensities secondary to severe epileptic seizure activity, potentially explaining the variation in reported presumptive diagnoses.^14^ Additionally, feline hippocampal and piriform lobe necrosis may occur secondary to severe epileptic activity, resulting in eosinophilic neuronal necrosis, astrogliosis and microglial reaction.^32^ A study by Wagner et al. reported that 83% of cats with hippocampal sclerosis had a history of epileptic seizures.^52^ Some cases of HN have been associated with autoantibodies against voltage‐gated potassium channel complex proteins and/or leucine‐rich glioma‐inactivated 1.^53^, ^54^ However, antibody status was not assessed in the present study.

In the current study, meningioma was highly suspected in approximately 58% (18/31) of cats with presumptive extra‐axial lesions, representing 11% (18/167) of cats with SL and approximately 4% (18/432) of the entire study population. This prevalence in cats with SL is slightly higher than the 7% (5/70) of cats with SE reported by Wagner et al.^52^ but lower than the 38% (17/45) described by Schrifl et al. in cats with presumptive neoplasia.^1^ Another study found that meningioma was responsible for SE in two of 15 (13%) cats.^7^ While meningiomas have historically been considered the most common intracranial neoplasm in cats, comprising approximately 33%‒58% of reported cases,^55^, ^56^ our findings suggest a lower prevalence. Although this study did not include histopathological confirmation, MRI remains a highly effective modality for detecting brain tumours in feline patients, with meningiomas being the most common differential diagnosis for extra‐axial masses with contrast enhancement and dural tail^24^.

One limitation of this study is that histopathological confirmation was unavailable, primarily because imaging studies were submitted to the teleradiology company VetCT prior to any biopsy procedures. As such, the reported findings represent imaging‐based presumptive aetiologies only. This limitation reflects real‐world clinical practice, and in such cases, additional diagnostic procedures, including CSF analysis or tissue sampling, are recommended to support or refine the diagnosis. The lack of CSF analysis in the majority of cases represents a noteworthy limitation, particularly given its diagnostic utility, alongside histopathology, in distinguishing infectious and immune‐mediated diseases from neoplastic processes, or in instances where subtle inflammatory changes may not be detectable on MRI.^28^ In this study, CSF analysis was available in only 15 cases, all of which yielded unremarkable results. This is likely to reflect common clinical practice, in which advanced imaging is often performed prior to CSF collection. Therefore, the true prevalence of presumed inflammatory encephalopathies may have been underestimated. MRI findings alone cannot always reliably distinguish neoplastic from inflammatory lesions, although they provide valuable diagnostic insight.^24^, ^28^, ^57^ However, prior research has shown that CSF analysis in cats with unremarkable MRI or with hippocampal signal changes are frequently within normal limits.^58^ Cats with MRI abnormalities, including lesions within the frontal sinus with a presumed intact cribriform plate and/or mild ventriculomegaly, for which an underlying cause could not be definitively identified were not excluded from this study. This was because they were not normal, and an intra‐axial component could not be entirely ruled out without histopathology. However, as these changes were considered unlikely to be the primary cause of epileptic seizures, these cases were categorised as ‘unknown relevance’ rather than being classified as IE or EuA.

For the purposes of this study, cats with imaging findings suggestive of a presumed metabolic, nutritional (thiamine deficiency) or toxic aetiology, were included as well. Such an approach was chosen as MRI findings in feline patients may reveal structural brain changes suggestive of these conditions. This classification more accurately reflects real‐world clinical scenarios, in which MRI findings may raise suspicion for metabolic or nutritional disorders and prompt further diagnostic testing. Furthermore, in the absence of histopathological confirmation, certain degenerative conditions can mimic metabolic disorders due to their characteristic bilateral and symmetrical MRI distribution patterns.^19^

Inclusion of cats with potentially isolated epileptic events reflects clinical practice, where advanced imaging is pursued without awaiting epileptic seizure recurrence, particularly when reactive seizures are deemed less likely. Patient signalment (e.g., age) and specific abnormalities identified following a neurological examination are important factors influencing the decision to perform an MRI after a single seizure and inclusion of these cases is reflective of routine diagnostic decision making in both primary care and referral settings. It is possible that some cases categorised as EuA or IE may have experienced epileptic seizures secondary to metabolic or toxic disorders that were neither identified nor documented. This could potentially contribute to an overrepresentation of these groups, given that cats in both categories show no visible changes on MRI. On the other hand, some cats classified as EuA based on non‐specific seizure descriptions may have belonged to the IE cohort. This challenge was exacerbated by the retrospective nature of the study, which relied on keyword‐based searches of MRI submissions to VetCT. Often, these submissions were accompanied by only brief or incomplete histories, lacking detailed epileptic seizure descriptions or containing only keywords (e.g., ‘seizure(s)’, ‘convulsion(s)’, etc.). Consequently, some cases labelled simply as ‘seizure(s)’, may have encompassed non‐epileptic paroxysmal events that were misclassified. Cats were excluded when additional clinical details suggested that the observed episodes were more consistent with vestibular dysfunction rather than epilepsy. This exclusion process reduced the initial dataset from 1465 brain MRI studies to 432 cases. However, given the potential for misinterpretation of seizure‐like episodes by both owners and primary practitioners, it is possible that some reported ‘seizures’ in the VetCT database were initially misclassified. A larger population was established to account for the inherent risk of misinterpreting epileptic seizures, a general challenge that extends not only to owners and general practitioners but also to experienced board‐certified specialists.

Furthermore, the study did not differentiate between cases referred by primary care veterinarians and those submitted by neurology specialists. While the recognition of generalised epileptic seizures is generally comparable between specialists and first‐opinion practitioners, the same cannot be said for focal epileptic seizures or other paroxysmal episodes/events.^59^ Even experienced specialists may encounter difficulties in accurately classifying such episodes, underscoring the inherent complexity of epilepsy diagnosis. Electroencephalography (EEG), while valuable in seizure classification, is not routinely used in veterinary medicine due to technical, accessibility and interpretation challenges. EEG data were not available for this study. Last, seizure type descriptions, treatment outcomes, response to therapy and follow‐up data were not analysed, as these factors fell outside the primary scope of this study.

In conclusion, this retrospective imaging‐based study demonstrates that two‐thirds of cats with suspected epileptic seizures have NLs on MRI. These results suggest that in cases where advanced diagnostics such as MRI are not feasible due to financial or logistical constraints, empirical medical management may still be a reasonable option, given the likelihood of an unremarkable MRI in a substantial proportion of affected cats.

## AUTHOR CONTRIBUTIONS

*Study idea*: Holger A. Volk and Julien Labruyère. *Data collection*: Julien Labruyère. *Data analysis and data interpretation*: Anna Knebel and Sebastian Meller. *Preparation of the first draft*: Anna Knebel, Sebastian Meller and Holger A. Volk. All the authors have read and approved the final draft of the manuscript.

## CONFLICT OF INTEREST STATEMENT

None of the authors of this paper have a financial or personal relationship with other people or organisations that could inappropriately influence or bias the content of the paper.

## FUNDING INFORMATION

The authors received no specific funding for this study.

## ETHICS STATEMENT

Owners provided informed consent for the clinical data of their cats to be used for research purposes. No further ethical requirement was needed under the local rules and regulations of the University.

## Supporting information



Supporting Information: vetr70737‐sup‐0001‐SupMat‐Table‐2.docx

## Data Availability

Data are available from the corresponding author upon reasonable request.
