# Supplementary material for: Epidemiological distribution of MRI‐based presumptive imaging diagnoses in a large general population of cats with suspected epileptic seizures
Source: Vet Rec. 2026 May 9;199(10):e445–52. doi: 10.1002/vetr.70737 (PMC13768726; doi:10.1002/vetr.70737)
Supplement: Supplementary file 1 [file VETR-199--s001.docx]

**Supplementary material:**

**Table 2:**

| **Information regarding further examinations and tests all cats (n=432)** | | | |
| --- | --- | --- | --- |
| **Performed examinations, tests, and analyses** | **Group 1: Cats with available data in regard to an examination and findings were mentioned as `unremarkable´**  **(number and percentage of these patients are given as `n=´ and in %)** | **Group 2: Cats with available data in regard to an examination and findings were mentioned as `abnormal´ (number and percentage of these patients are given as `n=´ and in%)** | **Group 3: Cats with unavailable data in regard to an examination (number and percentage of these patients are given as `n=´ and in %)** |
| General examination | 105 cats (24%) | 47 cats (11%) | 280 cats (65%) |
| Neurological examination | 128 cats (30%) | 101 cats (23%) | 203 cats (47%) |
| Blood tests (complete blood cell count, biochemical profile (liver biochemistry profile and if given serum bile acids or ammonia, kidney biochemistry profile and electrolytes) | 111 cats (26%) | 63 cats (14%) | 258 cats (60%) |
| Urinalysis | 11 cats (3%) | 4 cats (1%) | 417 cats (96%) |
| FIV, FELV, *Toxoplasma gondii* testing (blood samples) | 40 cats (9%) | 9 cats (2%) | 383 cats (89%) |
| CSF analysis | 15 cats (3%) | - | 417 cats (97%) |
